# Characterization of Global Research Trends and Prospects on Prone Positioning in Respiratory Failure: Bibliometric Analysis

**DOI:** 10.2196/67276

**Published:** 2025-06-20

**Authors:** Rong Lei, Feng Yue, Chaofu Yue, Zihan Zhang, Xian Huang, Qiaolin Li, Zhigang Yang, Rong Li, Keyi Zhao, Mei Yang

**Affiliations:** 1 The Qujing NO.1 People's Hospital, No. 1 Yuanlin Road, Qilin District, Qujing City, 655000, China, 86 15587199022

**Keywords:** acute respiratory distress syndrome, respiratory failure, acute respiratory failure, ARF, bibliometric analysis, critical care, bibliometrics, lungs, prone positioning, COVID-19, SARS-CoV-2, coronavirus, infectious, pulmonary, pandemic

## Abstract

**Background:**

Prone positioning has emerged as a crucial intervention in managing acute respiratory failure, especially in acute respiratory distress syndrome and patients with COVID-19. Given the increasing interest in this field, it is important to characterize global research trends and key contributors to identify future research directions.

**Objective:**

This study aimed to analyze global research trends, collaboration networks, and research hotspots related to prone positioning in respiratory failure through a comprehensive bibliometric analysis.

**Methods:**

Bibliometric analyses were conducted using CiteSpace and Biblioshiny software on publications up to December 31, 2023, from the Web of Science Core Collection, focusing on prone positioning in respiratory failure.

**Results:**

A total of 1263 research articles were identified, published in 50 countries by numerous institutions. The United States, France, and Germany contributed the most publications, with the United States producing 21.9% (275/1263) of the total. Key authors such as Claude Guerin and Luciano Gattinoni were identified as major contributors to the field. Keyword co-occurrence analysis revealed the dynamic nature of prone positioning research in respiratory failure. It highlighted protective ventilation and COVID-19-related acute respiratory distress syndrome as emerging hotspots, indicating a shift in focus during the pandemic.

**Conclusions:**

This study revealed a rapidly growing body of literature on prone positioning in respiratory failure, especially in the context of COVID-19. The findings underscore the importance of further multicenter clinical trials to validate current practices and refine treatment protocols. In addition, the application of prone positioning in non-intubated patients represents a potential future research direction.

## Introduction

Prone positioning, defined as a posture in which the patient lies face down with the chest and abdomen in contact with the surface and the back facing upwards, is commonly used in clinical settings for respiratory support and surgical procedures. Recently, prone positioning has gained significant attention as a therapeutic intervention, particularly in the management of acute respiratory failure (ARF) [1]. Its effectiveness is rooted in the interplay between body positioning and pulmonary mechanics, a relationship that has been explored extensively in recent decades. Historically, respiratory management in patients with ARF has focused on methods to enhance oxygenation and reduce complications [2].

In 1974, Mellins et al [3] investigated how physical therapies and body positioning, including the prone positioning, could influence lung volumes and facilitate better secretion clearance in pediatric patients with pulmonary diseases, supporting the notion that posture significantly impacts respiratory mechanics. Subsequently Bryan [4] offered a critical perspective on the mechanical implications of body positioning, and suggested that the supine position could exacerbate loss of lung volume in dependent regions due to gravitational effects on the diaphragm, leading to diminished ventilation and potential airway closure. Over the following decades, the role of prone positioning in improving oxygenation in patients with acute ARF was further investigated. In 2001, Gattinoni et al [5] conducted a randomized clinical trial on prone positioning in patients with acute respiratory distress syndrome (ARDS). While the study showed improvement in oxygenation, it did not demonstrate a significant survival benefit; however, it laid the groundwork for further investigation. The pivotal PROSEVA trial in 2013, conducted by Guérin et al [6], demonstrated that early and prolonged prone positioning significantly reduced 28-day and 90-day mortality in severe patients with ARDS, leading to its recommendation as a standard practice for managing ARDS.

The COVID-19 pandemic has renewed attention to prone positioning, not only for intubated patients but also, notably, for awake, nonintubated patients experiencing hypoxemia [7]. Studies during the pandemic have confirmed that prone positioning can improve oxygenation in patients with COVID-19 and can reduce the need for endotracheal intubation, which is confirmed by computed tomography scan and electrical impedance tomography [8]. The cumulative findings from these studies have shaped current respiratory care practices, where prone positioning is now routinely used in ARF management. This evolution in understanding highlights the interplay between pulmonary mechanics, perfusion distribution, and body positioning, establishing the prone intervention as a valuable tool in clinical practice [9]. As the evidence supporting prone continues to grow, it becomes increasingly important to assess its broader impact on clinical practice, thereby guiding future research and refining its application across diverse clinical settings.

Bibliometric analysis provides a quantitative approach to assessing the development and dissemination of knowledge within a particular field [10]. By mapping the trajectory of research on prone positioning and respiratory failure, it is possible to identify key trends, influential studies, and gaps in the existing literature. This, in turn, offers valuable insights into the evolution of clinical practice and the global impact of research efforts [11]. Previous bibliometric studies in respiratory failure have addressed various topics, none have specifically focused on prone positioning in respiratory failure [12,13]. This study aims to fill this gap by performing a comprehensive bibliometric analysis of the existing literature on prone positioning and its application in respiratory failure. Such insights are essential for guiding future research directions, promoting international collaboration, and ensuring that the benefits of prone positioning are fully realized in diverse clinical settings.

## Methods

### Data Source and Search Strategy

The data for this bibliometric analysis were sourced from the Web of Science Core Collection (WoSCC). A comprehensive search was conducted on August 21, 2024, using the following search strategy: “prone positioning” AND (“acute respiratory failure” OR “ARDS” OR “respiratory failure” OR “acute respiratory distress syndrome”). This strategy was designed capture studies on prone positioning in respiratory failure, using both general and specific terms. The initial search identified a total of 2143 records.

### Inclusion and Exclusion Criteria

To refine the dataset for bibliometric analysis, specific inclusion and exclusion criteria were applied. Initially, 108 records from the year 2024 were excluded to ensure the analysis focused on more established literature, resulting in 2035 articles. The dataset was further refined by excluding the following types of publications: 360 review articles, 126 meeting abstracts, 121 editorial materials, 114 letters, 43 proceeding papers, 5 corrections, and 3 early access articles. These exclusions were made to retain only original research articles, which are more likely to contribute directly to the understanding and development of prone positioning in respiratory failure. After applying these criteria, 1263 articles were selected for subsequent analysis, and no duplicates were found.

### Data Cleaning and Preprocessing

To ensure the accuracy and consistency of our dataset, we conducted a comprehensive data cleaning and preprocessing procedure. This process involved the removal of duplicate records, identified through digital object identifiers and study titles. We used the “biblioshiny” package in RStudio to standardize author and institutional names, consolidating any variations. In addition, the merge function was used to address synonyms, aliases, and singularor plural discrepancies in keywords. Noninformative keywords, such as “care,” “failure,” and “trial,” were removed using the remove function.

### Bibliometric Analysis

Our bibliometric analysis followed a broad-to-specific framework, including an overview, countries or districts, authors or institutions, journals or most cited publications, keywords and trends. The collected data were imported into Biblioshiny (R version 4.3.3; Institute for Statistics and Mathematics, Vienna, Austria) [14], CiteSpace (6.3.R1; Drexel University, Philadelphia, PA, the United States for social network analysis and Burst detection [15], and Microsoft Office Excel 2016 for further analysis.

Biblioshiny is primarily used to visualize the analysis of global publication trends, countries, sources, and influential documents [16]. A range of bibliometric indicators are analyzed through biblioshiny to assess the output of countries, and journals. The number of publications was used to quantify productivity, while TC reflected academic influence. Local citations were examined to assess impact within specific subject areas. To evaluate the impact of sources, we assessed theh-index, g-index, and m-index [17]. The h-index provides a balanced view of productivity and citation impact, while the g-index emphasizes highly-cited articles. The m-index accounts for publication longevity, normalizing for years of activity. CiteSpace is a Java-based software developed by Chen for bibliometric analysis, supporting knowledge mining and data visualization. We used it to conduct author cocitation analysis, institution cocitation analysis, and keyword co-occurrence analysis. Visual network graphs were used to illustrate the relationships among nodes, where larger node sizes indicate a greater number of publications within a particular research area [18]. We also conducted keyword burst analysis to identify emerging research themes over time [19].

## Results

### Search Results

**Figure 1.**
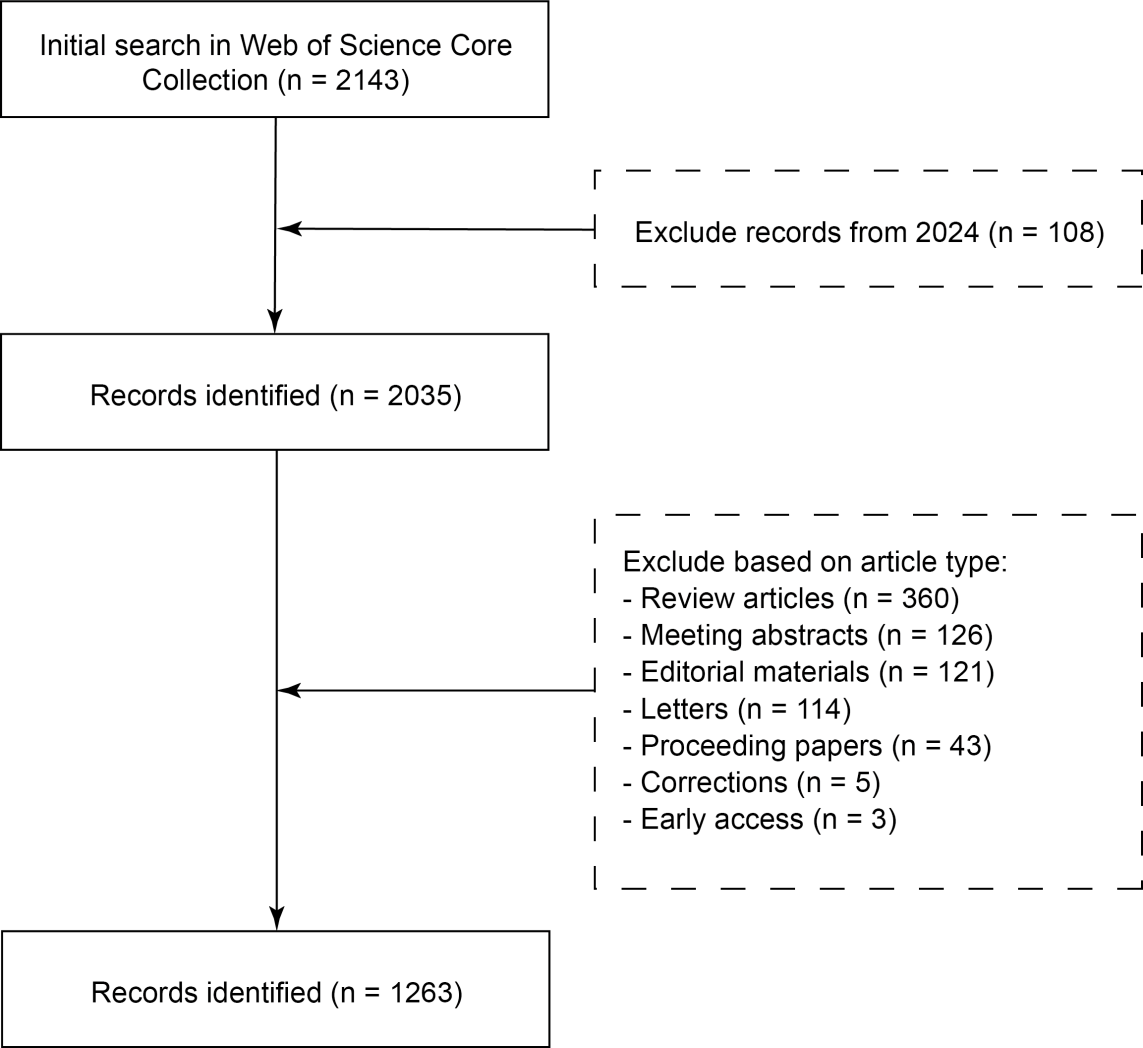
Record identification and selection.

A total of 2143 publications were identified from WoSCC. After excluding records from 2024 and filtering based on article type. Finally, 1263 studies were identified for bibliometric analysis (Figure 1).

### Global Publication Trends

**Figure 2.**
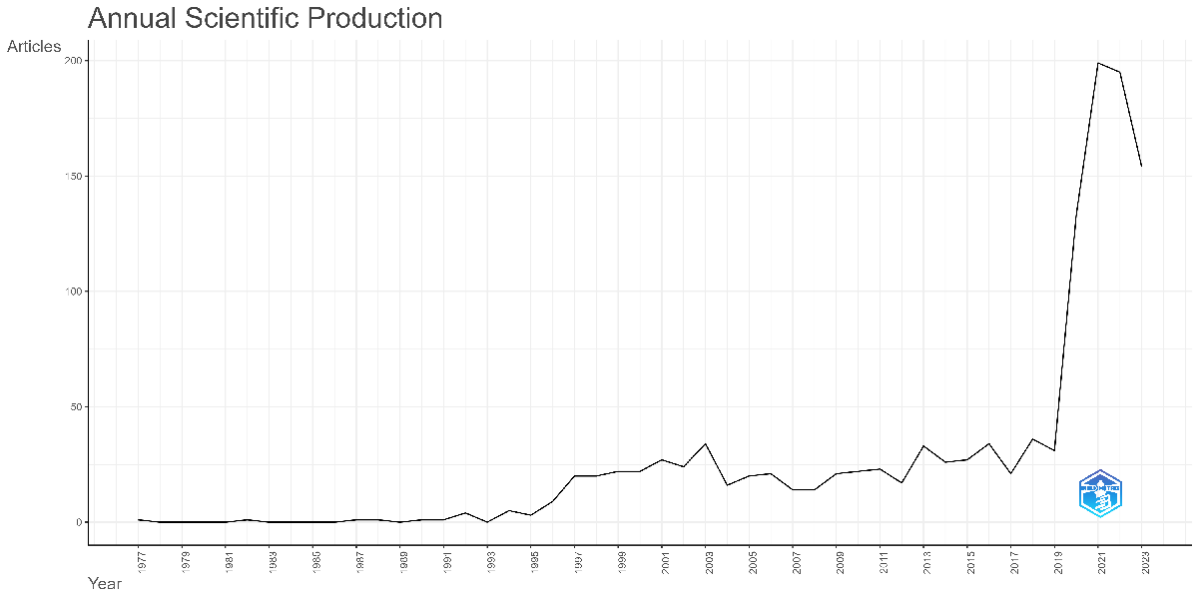
Annual scientific production. This figure shows the annual number of publications on prone positioning in respiratory failure from 1977 to 2023. The first publication appeared in 1977, with only 1 to 5 articles per year before 1995. From 1995 to 2018, there was a gradual increase in publications. A significant rise in the number of publications occurred from 2019 to 2023, with 199 publications in 2021.

The first publication on prone positioning in respiratory failure appeared in 1977, reporting that prone positioning significantly improves arterial oxygenation, allowing for reduced inspired oxygen concentrations in patients with ARF. In addition, there was only 1 to 5 publications per year before 1995. From 1995 to 2018, the number of publications showed a slow upward trend, whereas between2019 and 2023, there was a sharp increase, beginning with 31 publications in 2019 and peaking at 199 publications in 2021 (Figure 2).

### Countries and Districts

A total of 57 countries contributed to publications on prone positioning in respiratory failure. The United States was the leading contributor, with 275 publications (275/1253, 21.9% of the total), followed by France with 137 publications (137/1253, 10.9%), and Germany with 108 publications (108/1253, 8.6%). Italy contributed 97 publications (97/1253, 7.7%), and China contributed 92 publications (92/1253, 7.3%). Other major contributors included Canada (44/1253; 3.5%), Japan (39/1253; 3.1%), Spain (39/1253; 3.1%), the United Kingdom (37/1253; 3.0%), and India (27/1253; 2.2%).

In terms of citation impact, the United States had the highest total number of citations (10,146) with an average of 36.9 citations per document. France followed with 9229 citations and an average of 67.4 citations per document. Notably, Canada, despite contributing only 3.5% (44/1253) of the publications, had the highest average citations per document, reaching 132.5.

**Figure 3.**
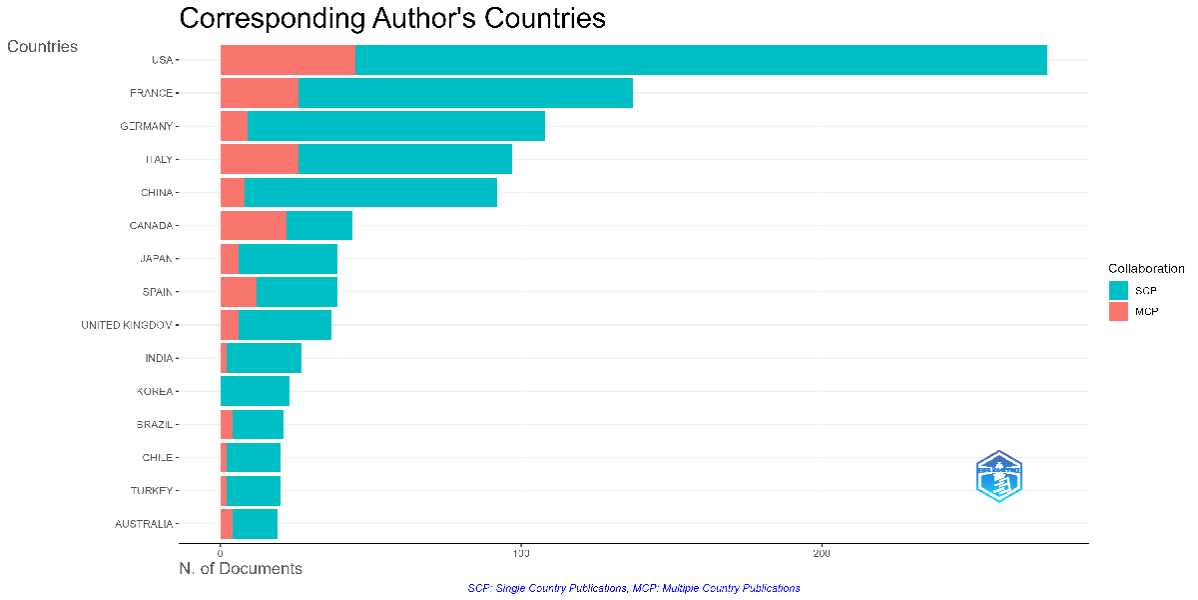
Corresponding author’s country distribution. This figure presents the distribution of single-country publications and multiple-country publications by country.

The analysis of corresponding authors’ countries revealed varying patterns of domestic and international collaboration (Figure 3; Sheet 1 in Multimedia Appendix 1). The United States had the highest total number of publications, with 230 single-country publications and 45 multiple-country publications (MCPs), resulting in an MCP rate of 16.4% (45/275). Although Canada had a relatively lower publication count (44 articles), it exhibited the highest MCP rate among the top contributing countries, with 50% of its publications involving international collaboration. By contrast, India had the lowest MCP rate at 7.4% (2/27), reflecting a higher proportion of domestically authored publications.

### Authors and Institutions

**Figure 4.**
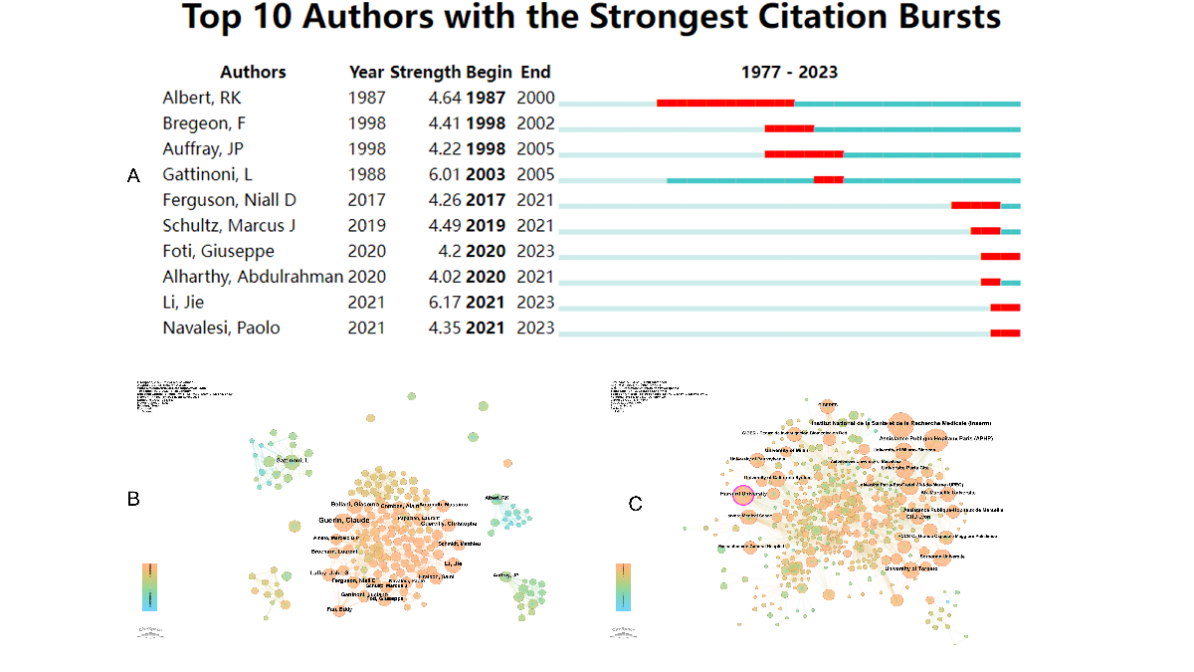
Authors and institutions. (**A**) Top 10 Authors with the Strongest Citation Bursts. This panel displays the top 10 authors with the strongest citation bursts in the field of prone positioning and respiratory failure. The red line segments represent the time periods of citation bursts, while the burst strength indicates the intensity of citation activity, with higher values reflecting stronger bursts. (**B**) Co-authorship network Visualization. This panel visualizes the co-authorship network in prone positioning research. Nodes represent authors, and edges between nodes represent co-authorship relationships. The network highlights key authors and their collaboration patterns over time.(**C**) Network of Co-authors’ Institution Visualization. This panel illustrates the co-authorship network at the institutional level. Nodes represent institutions, and edges between them reflect collaborative publishing efforts. The network highlights prominent institutions and their involvement in global research initiatives on prone positioning and respiratory failure.

A total of 553 authors contributed to publications on prone positioning and respiratory failure. Albert et al [20-23] published 6 articles and experienced a notable citation burst beginning in 1987 and continuing until 2000, with a burst strength of 4.64 (Figure 4A). Papazian et al [24,23] also experienced a citation burst from 1998 to 2005, with a strength of 4.22. Gattinoni demonstrated the highest citation burst strength of 6.01, occurring between 2003 and 2005. With 10 publications, Gattinoni has been a leading figure in this field since 1988, particularly in research on ARDS and prone positioning. More recently, Ehrmann et al [25] and Cammarota [26] have shown emerging influence, with citation bursts beginning in 2021 and continuing through 2023, and burst strengths of 6.17 and 4.35, respectively. Network analysis identified Claude Guérin as the most prolific and central author, with 16 publications and a centrality score of 0.03, underscoring his influential role in this research domain (Sheet 2 in Multimedia Appendix 1). Guérin’s contributions have been especially significant since 2006 (Figure 4B). In the early stages of the field, authors such as Gattinoni et al [27], and Michelet et al [23] conducted research in relatively isolated clusters. However, since the onset of the COVID-19 pandemic, collaboration among researchers has increased notably, reflecting a shift toward more integrated global research.networks. Institut National de la Santé et de la Recherche Médicale and Assistance Publique–Hôpitaux de Paris, with 82 and 70 publications respectively, rank among the top institutions contributing to research on prone positioning and respiratory failure (Figure 4C). Harvard University leads with 51 publications and exhibits the highest centrality score (0.15), indicating its pivotal role in connecting diverse research clusters within the global collaboration network. Other institutions, such as Leipzig University and the University of Milan, also demonstrate relatively high centrality values (0.09 and 0.08, respectively), suggesting that they serve as important bridges across international research communities (Sheets 3 in Multimedia Appendix 1). An emerging contributor, Southeast University in China, reflects the growing involvement of Chinese institutions in this research domain.The analysis further reveals distinct collaboration patterns, with some institutions predominantly engaging in domestic (intracountry) collaborations, while others—such as Harvard University—participate extensively in international research partnerships, advancing global progress in the study of prone positioning.

### Journals and Most Cited Publications

**Figure 5.**
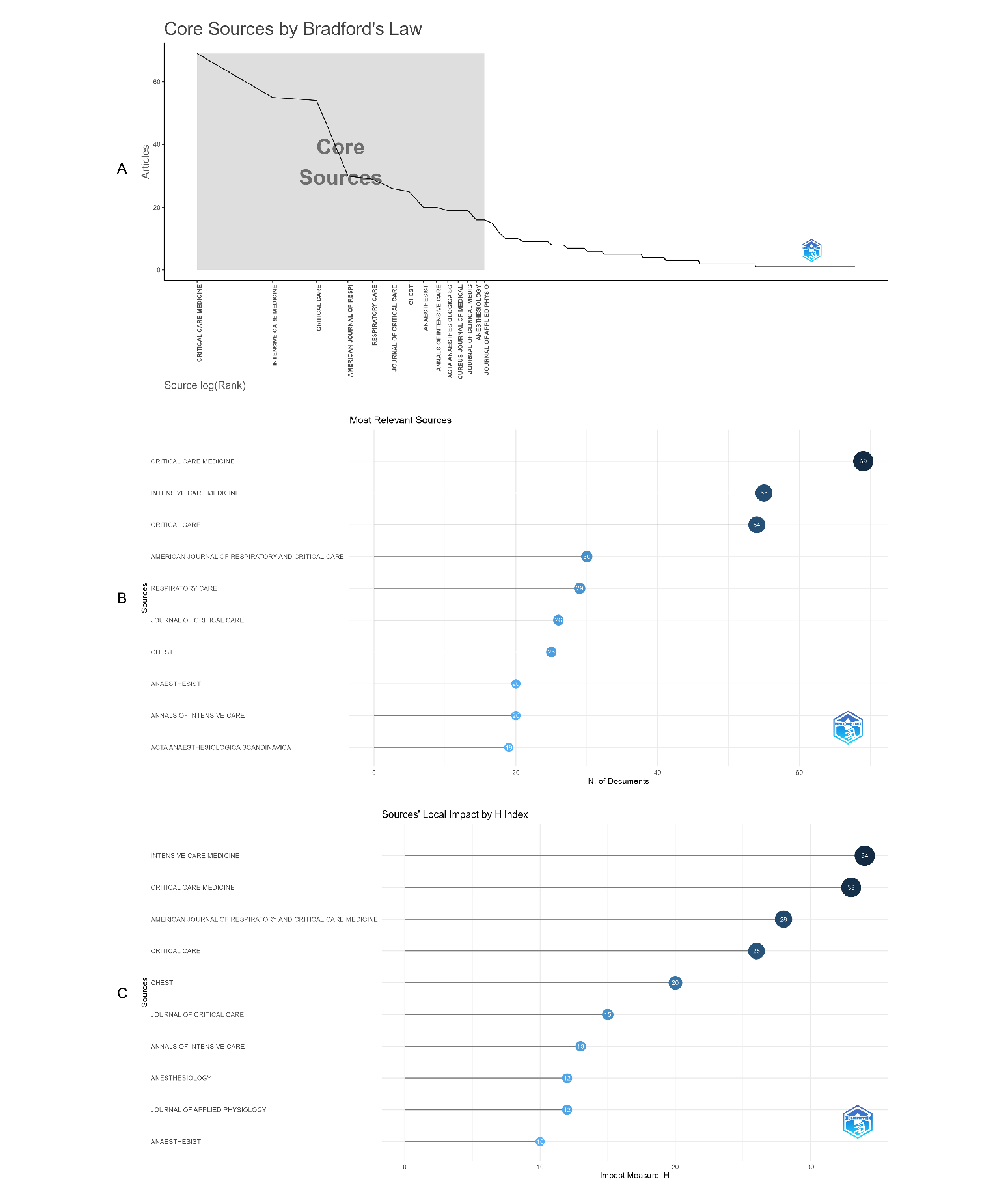
Authors and institutions. (**A**) Bradford Law bibliometrix. (**B**) Most relevant journals. (**C**) Impact index of journals.

Bradford’s Law provides a framework for quantifying the distribution of articles across journals within a specific research field. It posits that publications can be grouped into a core set of journals contributing the largest number of articles, followed by successive zones with decreasing productivity. According to Bradford’s Law Analysis, 13 journals were identified as core journals, The leading journal, *Critical Care Medicine*, has the highest frequency with 69 articles, followed by *Intensive Care Medicine* (55 articles), and *Critical Care* (54 articles). These journals constitute the core outlets for high-impact and frequently cited research on prone positioning and respiratory failure (Figure 5A). Further analysis of relevant sources confirmed *Critical Care Medicin*e as the most productive journal, followed closely by *Intensive Care Medicine* and *Critical Care*. *The American Journal of Respiratory and Critical Care Medicine* also ranked highly, with 30 articles published in this area (Figure 5B). In terms of source impact, *Intensive Care Medicine* demonstrated the highest h-index of 34, along with the highest TC of 5925. This was closely followed by *Critical Care Medicine* (h-index 33, TC 4307) and *The American Journal of Respiratory and Critical Care Medicine* (h-index 28, TC 4726) (Figure 5C).

High values across h-index, g-index, and m-index indicate that these journals exhibit both substantial productivity and significant impact in the field of respiratory failure, particularly concerning prone positioning (Sheet 3 in Multimedia Appendix 1). *Critical Care Medicine* and *Intensive Care Medicine* had comparable m-index values of 1.179 and 1.172, respectively, suggesting sustained academic influence since their emergence in the mid-to-late 1990s. Their consistent growth in citations and publication output reflects their central role in disseminating research in this area (Sheet 4 in Multimedia Appendix 1). Despite its relatively recent entry into the field (established in 2015), *Annals of Intensive Care* recorded the highest m-index (1.3), indicating rapid growth in influence. In contrast, older, more established journals such as *CHEST* and *The Journal of Applied Physiology* have shown steadier but slower growth in impact over time.

The most cited publication in this analysis is the study by Bellani et al [28], published in *JAMA*, which received 3240 TCs, with an average of 360 citations per year (TC/Y) and a normalized TC score of 23.25 (Table 1). This pivotal study presents large-scale data from the LUNG SAFE study, offering critical insights into the epidemiology, management, and outcomes of ARDS. Closely following is the work by Guérin et al (2013), published in *The New England Journal of Medicine*, with 2423 TC, a TC/Y of 201.92, and a normalized TC of 12.85. This landmark clinical trial established the efficacy of prone positioning in improving survival rates among patients with severe ARDS, and it fundamentally altered international treatment guidelines. A more recent publication by Schmidt et al (2021), appearing in *Intensive Care Medicine*, has already accumulated 476 citations, with a remarkable TC/Y of 119.00 and a normalized TC of 22.80. The rapid accumulation of citations reflects the high relevance of this study, particularly in the context of the COVID-19 pandemic, during which the management of respiratory failure and ARDS has become a global research priority.

**Table 1.** The most cited publication by top 10-record.

Rank	Title	TC^a^	TC per year	Normalized TC
1	Epidemiology, patterns of care, and mortality for patients with acute respiratory distress syndrome in intensive care units in 50 countries	3240	360.00	23.25
2	Prone positioning in severe acute respiratory distress syndrome	2423	201.92	12.85
3	Efficacy and economic assessment of conventional ventilatory support versus extracorporeal membrane oxygenation for severe adult respiratory failure (CESAR): a multicentre randomised controlled trial	2301	143.81	15.63
4	Surviving sepsis campaign: international guidelines for management of severe sepsis and septic shock, 2012	1426	118.83	7.56
5	Surviving sepsis campaign: international guidelines for management of severe sepsis and septic shock: 2012	933	77.75	4.95
6	An official american thoracic society/European Society of intensive care medicine/society of critical care medicine clinical practice guideline: mechanical ventilation in adult patients with acute respiratory distress syndrome	929	116.13	13.84
7	Effect of prone positioning on the survival of patients with acute respiratory failure	733	30.54	14.28
8	Extracorporeal membrane oxygenation for ARDS in adults	575	41.07	12.34
9	The concept of “baby lung”	505	25.25	5.91
10	Clinical characteristics and day-90 outcomes of 4244 critically ill adults with COVID-19: a prospective cohort study	476	119.00	22.80

a TC: total citation.

### Co-Occurring Author Keywords

**Figure 6.**
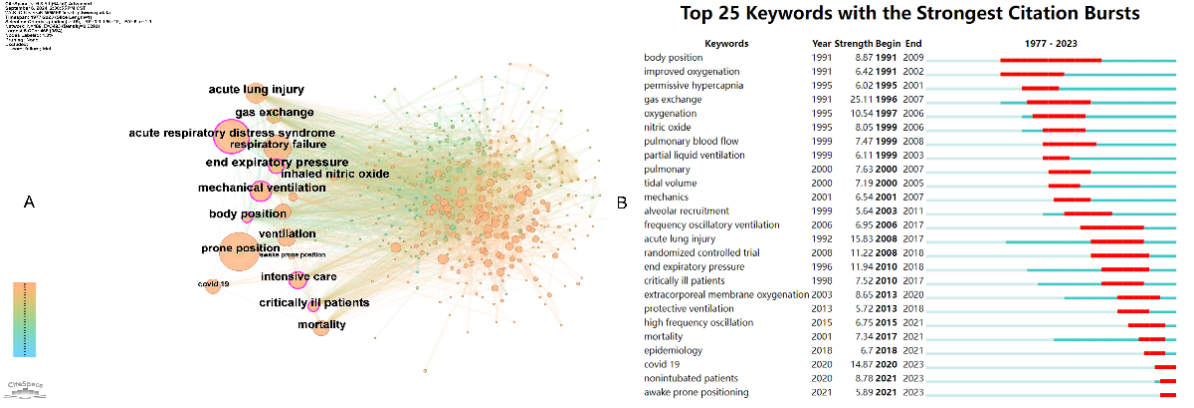
Co-occurring author keywords analysis. (**A**) Co-occurring author keywords network Visualization. This panel visualizes the network of co-occurring author keywords related to prone positioning and respiratory failure. Nodes represent individual keywords, and edges between nodes indicate the co-occurrence relationships. The network highlights the most frequently mentioned and connected terms in the research field.(**B**) Top 25 Keywords with the Strongest Citation Bursts. This panel presents the top 25 keywords with the strongest citation bursts, reflecting trends in the research over time. The red line segments represent the time periods of citation bursts, while the burst strength indicates the intensity of citation activity, with higher values reflecting stronger bursts.

In the analysis of co-occurring author keywords related to prone positioning and respiratory failure, several critical terms emerged as focal points, reflecting the research trends and clinical focus areas over the years. Prone position (count: 592 and centrality: 0.08), was the most frequently occurring keyword, consistently central to discussions on improving oxygenation and outcomes in patients with ARDS and respiratory failure (Figure 6A). ARDS (count: 475, centrality: 0.1) is the second most frequent keyword, highlighting the prominence of ARDS in the context of respiratory failure, particularly in mechanically ventilated patients (Sheet 5 in Multimedia Appendix 1). The co-occurrence of mechanical ventilation (count: 292 and centrality: 0.12) further emphasizes the critical relationship between mechanical support strategies and the management of ARDS and respiratory failure. The inclusion of extracorporeal membrane oxygenation (count: 111) and inhaled nitric oxide (count: 79) among the top keywords reflects the exploration of advanced therapies for refractory hypoxemia, often in conjunction with prone positioning. Notably, COVID-19 (count: 58) and awake prone positioning (count: 18) have emerged as recent significant keywords, reflecting the shift in clinical focus due to the pandemic and the adaptation of prone positioning in nonintubated, awake patients to improve oxygenation without invasive mechanical ventilation.

Based on the keywords burst analysis, the top 25 keywords revealed clear trends in the evolution of research on prone positioning and respiratory failure (Figure 6B).

#### Early Stage (around 2000)

During this period, keywords such as body position and gas exchange were prominent. These terms suggest that the research focus was centered on understanding the pathophysiology of ARDS and improving treatment strategies—particularly by optimizing mechanical ventilation. Researchers aimed to enhance survival rates among patients with ARDS by refining ventilatory support techniques.

#### Middle Stage (around 2010)

In this phase, the emphasis shifted toward prone position and lung protective strategy, indicating a growing consensus on the physiological benefits of prone positioning and its role within lung-protective ventilation frameworks.

#### Recent Stage (around 2020)

In the most recent phase, keywords such as COVID-19, awake prone positioning, and nonintubated patients emerged strongly. These bursts reflect the global urgency in treating ARDS in the context of the COVID-19 pandemic. The renewed interest in prone positioning—particularly for awake, nonintubated patients—underscores its adoption as a noninvasive, life-saving intervention for COVID-19-related respiratory failure.

## Discussion

### Principal Findings

The bibliometric analysis of research on prone positioning in respiratory failure offers a comprehensive view of the evolution, global trends, key contributors, and future directions in this important clinical area. Prone positioning has evolved over the past 5 decades from a theoretical concept to a widely adopted therapeutic intervention [29,30]. Early observational studies demonstrated its potential to improve oxygenation, a finding later corroborated by clinical trials [2,3]. Subsequently, numerous cohort studies and randomized controlled trials, confirmed its efficacy in respiratory failure [31-34]. The landmark PROSEVA trial in 2013 demonstrated a significant reduction in 28-day and 90-day mortality rates when early and prolonged prone positioning was applied to patients with severe ARDS [6]. This study established prone positioning as a standard intervention in patients with severe ARDS, where conventional supine ventilation often fails to maintain adequate oxygenation. With the onset of the COVID-19 pandemic, prone positioning was rapidly adopted in hypoxemic respiratory failure related to COVID-19. Studies have shown that prone positioning can improve oxygenation not only in patients with mechanically ventilation but also in nonintubated, awake patients, suggesting a role in potentially delaying or preventing the need for invasive mechanical ventilation [35]. This trend underscores the importance of prone positioning in both traditional ARDS management and in the novel context of COVID-19, driving a surge in research output.

### National Contributions and Global Distribution

The United States leads global contributions to research on prone positioning in respiratory failure, accounting for 21.9% of the total publications (Table 2). The United States research output is not only substantial in volume but also impactful, as evidenced by its 10,146 citations and an average citation per document of 36.9. This aligns with the country’s leadership in critical care and respiratory research, as highlighted by key studies such as the LUNG SAFE study by Bellani et al [28]. The prominent role of European countries, including Germany, Italy, and France, reflects strong collaborations within the EU and international research networks, as exemplified by the PROSEVA trial [6]. Overall, the research landscape in prone positioning and respiratory failure is characterized by strong contributions from a mix of Western and Asian countries, with the United States, France, and Italy leading in terms of both quantity and impact of publications. The high average citation rates in countries like France, Canada, and the United Kingdom suggest that these nations produce particularly influential research, while emerging contributions from China and India highlight the increasing global interest and participation in this critical area of study [25,36-38].

**Table 2.** Distribution by country.

Rank	Country	Documents, n (%)	Citations, n	Average citations
1	United States	275 (21.9)	10146	36.90
2	France	137 (10.9)	9229	67.40
3..	Germany	108 (8.6)	2232	20.70
4	Italy	97 (7.7)	6008	61.90
5	China	92 (7.3)	1039	11.30
6	Canada	44 (3.5)	5831	132.50
7	Japan	39 (3.1)	348	8.90
8	Spain	39 (3.1)	1529	39.20
9	United Kingdom	37 (3)	3829	103.50
10	India	27 (2.2)	221	8.20

### Influential Authors and Scientific Impact

The analysis identified several key authors who have made foundational contributions to the field of prone positioning and respiratory failure. (Figure 4). In the early stage Albert et al have significantly advanced the understanding of the physiological effects and clinical benefits of prone positioning in managing ARF, particularly in ARDS. His work spans from animal models to human studies, elucidating mechanisms that improve gas exchange and reduce lung injury, and identifying that one of the key benefits of prone positioning was the alleviation of lung compression caused by the heart, which have shaped modern critical care practices [20-22,39]. The researcher from France, Auffray JP has played a significant role in advancing the understanding of prone positioning and its interaction with other treatments for ARDS. Auffray’s work, particularly from the late 1990s to the early 2000s, focused on exploring how the prone position, combined with adjunctive therapies like inhaled nitric oxide and pulmonary vasoconstrictors, could enhance oxygenation and improve clinical outcomes in patients with ARDS [23,24,40]. And his studies explored the variability in patient response to prone positioning, indicated that a short-term trial of prone positioning may not be sufficient to predict which patients will respond to the prone positioning therapy, showing the complexity of patient variability in ARDS management. He also examined the tomographic features of patients with ARDS, while prone positioning improved oxygenation in many cases, computed tomography scans could not reliably predict responders based on lung opacity [41,42].

Gattinoni L’s research [43] on ARDS spans over several decades, beginning in the late 1980s and continuing into the present. His pioneering work has evolved alongside advances in medical technology and clinical understanding of respiratory failure. His earliest influential study on prone positioning was published in 1988, where he investigated how body position could improve oxygenation in patients with ARDS [43]. Throughout the 1990s and 2000s, Gattinoni’s research expanded to establish the concept of the “baby lung,” which revolutionized the approach to mechanical ventilation by emphasizing the need to protect the small, aerated portion of the lungs in patients with ARDS. This work also laid the foundation for understanding ventilator-induced lung injury and its prevention through lung-protective strategies [44]. Gattinoni, L’s research into prone positioning, lung mechanics, and the differentiation between pulmonary and extrapulmonary ARDS continued well into the 2000s, with several landmark studies published in 2003. These studies helped shape modern ventilatory practices, emphasizing the importance of individualized ventilation settings and protective strategies [45-47]. His body of work remains highly influential in guiding the clinical management of ARDS, particularly in the context of critical care and mechanical ventilation.

Dr Claude Guérin, with the most publications and significant centrality in the coauthorship network, stands out as a pivotal figure in this area, particularly due to his work on the landmark PROSEVA trial, which demonstrated the survival benefit of prone positioning in patients with severe ARDS, provides robust evidence that prone positioning not only improved oxygenation but also led to significant reductions in 28-day and 90-day mortality rates [6]. Following the PROSEVA trial, Guérin continued to explore the physiological mechanisms and clinical applications of prone positioning. His subsequent studies addressed various aspects of prone positioning, including its effects on hemodynamics and respiratory mechanics [48]. Throughout his career, Guérin’s research has consistently focused on optimizing the use of prone positioning and understanding its physiological benefits. His work has shaped clinical guidelines and transformed the standard of care for patients with ARDS, particularly in the context of the COVID-19 pandemic. The breadth and depth of his research, ranging from clinical trials to mechanistic studies, underscore his pivotal role in advancing critical care practices for respiratory failure [49]. The citation burst of more recent authors like Li Jie and Navalesi Paolo indicates that research on prone positioning continues to evolve, with current studies exploring newer dimensions, such as its use in nonintubated patients during the COVID-19 pandemic [25,50].

### Institutional Landscape and Collaborative Networks

From an institutional perspective, several significant trends emerge that illuminate the global research landscape and collaboration dynamics in the field of prone positioning and respiratory failure. First, it is evident that highly productive authors are often affiliated with prestigious academic or medical institutions that drive global research efforts. For instance, Claude Guérin, one of the most prominent authors in this field, is affiliated with University of Lyon in France, which have contributed substantially to the field through several influential studies [6,51,52]. Institut National de la Santé et de la Recherche Médicale and Assistance Publique–Hôpitaux de Paris have been leaders in research output, while institutions such as Harvard University, Leipzig University, and the University of Milan exhibit high centrality, indicating their pivotal role in connecting global research networks [5,28,53-56]. These institutions often serve as research hubs due to their access to extensive clinical trial data and resources for conducting high-impact studies

Moreover, international collaboration is a hallmark of research in this field. European institutions, particularly those in France and Italy, demonstrate strong collaborative networks with North American institutions, such as Harvard University and the University of Toronto. These collaborations facilitate the sharing of clinical insights and promote the development of comprehensive guidelines, as evidenced by the high citation rates of multinational studies such as those by Guérin et al [6,49]. Chinese institutions, such as Southeast University, have shown a rapid increase in publication volume, signaling an emerging influence in global respiratory research [57,58]. These institutions drive forward clinical guidelines and research agendas, supported by strong international collaborations that enhance the global understanding of prone positioning in respiratory failure.

### Research Focus and Keyword Evolution

The evolution of research keywords provides valuable insight into the shifting focus and progression of scientific inquiry in this field [59]. By analyzing keyword trends over time, distinct phases of research development can be observed, revealing the core themes and emerging topics that have shaped the understanding of prone positioning as a therapeutic strategy in respiratory failure. Early studies primarily explored the effect of prone positioning on oxygenation in patients with ARDS, with mechanical ventilation being at the forefront of therapeutic interventions. During this period, research often emphasized the physiological benefits of prone positioning, particularly its ability to improve ventilation-perfusion matching and reduce shunt fraction [27,37,44,60,61]. In the middle phase, research shifted toward prone positioning and lung protective strategies. Prone positioning was increasingly recognized not only for its oxygenation benefits but also for its role in mitigating ventilator-induced lung injury [61-63]. The onset of the COVID-19 pandemic marked a new phase in the research on prone positioning, with keywords such as “COVID-19” and “awake prone positioning” from 2020 onwards. This period saw an explosion of research, as prone positioning was adapted for use in both intubated and nonintubated patients with COVID-19-induced respiratory failure [64-66]. Awake prone positioning, in particular, emerged as a critical intervention to delay or prevent intubation in hypoxemic patients with COVID-19. This technique was rapidly incorporated into clinical practice due to its simplicity, cost-effectiveness, and demonstrated efficacy in improving oxygenation without the need for mechanical ventilation [38,67].

### Research Challenges and Difficulties

Research on different body positions in the management of respiratory failure has evolved significantly over the years, with various positions being explored for their effects on oxygenation and ventilation. The supine position, while commonly used, can result in poor lung expansion in the dorsal regions due to the effects of gravity, leading to compression and collapse of these areas [68]. This can exacerbate ventilation inefficiency, particularly in patients with ARDS, where the lungs are already compromised by inflammation and fluid accumulation [27]. In contrast, prone positioning reverses the gravitational effects on the lungs. This position redistributes lung inflation along the gravitational axis, opening the collapsed dorsal lung regions while compressing the ventral regions. As a result, prone positioning enhances ventilation in the dorsal parts, which are typically underventilated in the supine position, thereby improving oxygenation. Yi Xin et al [69] demonstrated in a large animal model that prone positioning improves lung reinflation and recruitment, particularly in the dorso-caudal regions of the lung. Subsequent clinical applications have confirmed the effectiveness of prone positioning, and multiple clinical trials have been conducted to investigate its safety and efficacy in diverse patient populations [29]. However, despite its proven benefits, the application of prone positioning still faces several challenges. One major difficulty is the potential for complications associated with the physical maneuvering of patients into the prone position, which can result in inadvertent extubation or displacement of endotracheal tubes [70]. In addition, patients with certain comorbid conditions, such as obesity, cardiovascular instability, or spinal deformities, may not tolerate prone positioning as effectively. Obesity, in particular, complicates the effectiveness of prone positioning due to altered lung mechanics and reduced functional residual capacity, making it more difficult to achieve adequate lung recruitment during positioning [71]. Furthermore, prolonged prone positioning can increase the risk of pressure ulcers and facial injuries, especially in patients who cannot maintain the position without significant support [9]. These challenges underscore the need for careful patient selection, individualized treatment plans, and vigilant monitoring during the application of prone positioning, particularly in the presence of severe comorbidities. Recent studies suggest that alternative techniques, such as sequential lateral positioning and bed tilting, can also improve oxygenation and serve as viable substitutes for prone positioning [72]. Sequential lateral positioning involves alternating between supine and lateral positions to improve lung recruitment, redistribute ventilation, and enhance oxygenation in patients with ARDS. Adjusting the tilt of the bed also enhances alveolar recruitment and improves blood flow redistribution; however, tilting beyond 30° may cause overdistension and lead to hemodynamic instability [73].

### Recommendations for Future Work

Future research should focus on several key areas to advance the understanding and application of prone positioning in respiratory failure.

#### Awake Prone Positioning

While the benefits of prone positioning in intubated patients with ARDS are well-documented, more research is needed to understand its efficacy in nonintubated patients. The COVID-19 pandemic has prompted the use of awake prone positioning, but studies have reported inconsistent or even contradictory findings, highlighting the need for robust, multinational clinical trials to validate the results.

#### Personalized Prone Positioning Protocols

Research should explore whether personalized approaches to prone positioning, tailored to individual patient characteristics (eg, lung morphology, disease severity, lifestyle habits, nutritional status, BMI), can improve outcomes. Developing personalized prone positioning protocols could lead to more effective and targeted treatments for diverse patient populations.

#### Explore the Mechanisms Behind Prone Position

Future research should focus on understanding the physiological mechanisms underlying the effects of prone positioning on respiratory mechanics and cardiopulmonary interactions in patients with respiratory failure. Emerging technologies, such as electrical impedance tomography, offer opportunities for real-time monitoring of lung recruitment and ventilation-perfusion matching during prone positioning, which can further elucidate the physiological impact of this intervention.

#### Long-Term Outcomes

Most research to date has focused on short-term outcomes, such as oxygenation and survival rates. Future studies should examine the long-term impact of prone positioning on pulmonary function, quality of life, and psychological well-being in survivors.

#### International Collaboration

Given the complex, multidisciplinary nature of respiratory care, fostering international collaboration is crucial. Research efforts should prioritize cross-border partnerships to ensure that findings are generalizable across different health care settings and patient populations.

### Limitations

While this bibliometric analysis provides a comprehensive overview of research trends, it is not without limitations. First, the analysis was limited to publications indexed in the WoSCC, which may have excluded relevant studies from other databases such as Scopus, PubMed, and Embase. This could potentially lead to an underrepresentation of research from regions or countries where publication in these databases are more prevalent.

Second, the exclusion of non-English publications may have limited insights into research conducted in non-English-speaking regions, particularly in countries like China, Japan, and India, which are emerging contributors to prone positioning research. The reliance on English-language publications could introduce a bias, potentially underestimating the global scope of research in this field. Third, bibliometric analyses are inherently quantitative and do not assess the quality or clinical impact of the studies themselves. While citation metrics provide a proxy for influence, they do not always reflect the practical applicability of the research findings in clinical settings. High citation counts may sometimes be driven by factors unrelated to the study’s scientific rigor, such as controversies or methodological critiques.

### Conclusions

This bibliometric analysis highlights the expanding body of research on prone positioning in respiratory failure, with significant contributions from leading countries, institutions, and authors. The findings underscore the critical role of prone positioning in managing respiratory failure and improving patient outcomes, particularly in the context of the COVID-19 pandemic. Although significant progress has been made in this field, key gaps remain, particularly in understanding the application of prone positioning in nonintubated patients and the long-term outcomes. Future research should focus on addressing these gaps by exploring personalized treatment approaches, fostering international collaboration, and further investigating the physiological mechanisms underlying prone positioning. By building upon the existing evidence base, the medical community can ensure that prone positioning continues to evolve as a more effective and widely adopted intervention in the management of respiratory failure.

## Supplementary material

10.2196/67276 Multimedia Appendix 1 Sheets.

## Abbreviations

ARDS: Acute Respiratory Distress Syndrome
ARF: Acute Respiratory Failure
COVID-19: coronavirus disease 2019
MCP: Multiple-Country Publications
WoSCC: Web of Science Core Collection
